# Foveal Damage Due to Subfoveal Hemorrhage Associated with Branch Retinal Vein Occlusion

**DOI:** 10.1371/journal.pone.0144894

**Published:** 2015-12-14

**Authors:** Yuki Muraoka, Akitaka Tsujikawa, Ayako Takahashi, Yuto Iida, Tomoaki Murakami, Sotaro Ooto, Kiyoshi Suzuma, Akihito Uji, Nagahisa Yoshimura

**Affiliations:** 1 Department of Ophthalmology and Visual Sciences, Kyoto University Graduate School of Medicine, Kyoto, Japan; 2 Department of Ophthalmology, Kagawa University Faculty of Medicine, Kagawa, Japan; LV Prasad Eye Institute, INDIA

## Abstract

To investigate the functional and morphologic prognoses of eyes with subfoveal hemorrhage from acute branch retinal vein occlusion (BRVO), and to examine the effect of intravitreal ranibizumab injection (IVR) on these prognoses, we assessed 81 eyes with acute BRVO, of which 38 did not receive IVR [IVR(-) group], and 43 were treated with IVR [IVR(+) group] for macular edema. The foveal morphologic changes were examined via optical coherence tomography (OCT). At initial examination, 63 eyes exhibited subfoveal hemorrhage. At final examination, the defect lengths in the foveal external limiting membrane (ELM) and ellipsoid lines in these eyes were longer, and final VA was significantly poorer, compared with eyes without subfoveal hemorrhage. In comparisons between the final measurements in eyes with subfoveal hemorrhage in the IVR(-) and IVR(+) groups, while there were no differences in initial ocular conditions, final VA was significantly better in the IVR(+) group. The defects in the ELM and ellipsoid lines in the IVR(+) group were shorter than those of the IVR(-) group (*p* = 0.002 in both). Final VA was correlated with the defect lengths of foveal ELM and ellipsoid lines in both the IVR(-) and IVR(+) groups (both *p* < 0.001). In addition, the defect lengths of foveal ELM and ellipsoid lines were closely correlated with the duration of subfoveal hemorrhage (both *p* < 0.001). BRVO-associated subfoveal hemorrhage caused damage to the foveal photoreceptors, and visual dysfunction. However, IVR improved these prognoses, by accelerating the absorption of the subfoveal hemorrhage.

## Introduction

Acute branch retinal vein occlusion (BRVO) often accompanies macular edema (ME), serous retinal detachment, or retinal ischemia, and some of these features may lead to impaired visual function.[1,2,3,4] Anti-vascular endothelial growth factor (VEGF) agents, including ranibizumab, cause the rapid reduction of ME associated with BRVO, and can thus contribute to an improvement in visual acuity (VA).[5,6,7,8] In recent studies involving optical coherence tomography (OCT), visual function has reportedly been strongly correlated with the integrity of outer aspects of the foveal photoreceptor layer in eyes with BRVO.[9,10,11] Ota et al. reported that eyes with persistent ME after BRVO exhibited good VA when the integrity of the outer aspect of the foveal photoreceptor layer was preserved. On the other hand, in some cases VA was severely compromised even if complete resolution of the ME was achieved, when the foveal photoreceptors were damaged.[12]

Submacular hemorrhage secondary to age-related macular degeneration (AMD) or retinal arterial macroaneurysm (RMA) can often cause severe visual impairment.[13,14,15,16,17] Previous experimental studies suggest several mechanisms by which subretinal hemorrhage can damage the overlying photoreceptors, including iron toxicity, induction of subretinal fibrosis, and blockage of nutrient diffusion from the choroidal circulation.[18,19] Since Spaide et al. introduced OCT for the examination of subretinal hemorrhage, it has been recognized that acute BRVO sometimes accompanies the condition.[20] In a recent small case series, Muraoka et al. reported that subfoveal hemorrhage was not an uncommon feature of BRVO, and that it implied a poor visual prognosis. In eyes with acute BRVO, subfoveal hemorrhage may cause damage to the overlying outer aspect of the foveal photoreceptor layer, leading to visual dysfunction.[12]

In a large multicenter trial, Brown et al. recently reported that retinal hemorrhage cleared rapidly following treatment with ranibizumab, in eyes with BRVO. We speculate that ranibizumab does not facilitate the absorption of the intraretinal hemorrhage directly, but rather, that its suppressive effect on new bleeding seemingly accelerates the absorption. Considering this mechanism, ranibizumab may accelerate the absorption of subfoveal hemorrhage, which might subsequently contribute to mitigating damage to the overlying foveal photoreceptors. To investigate the impact of subfoveal hemorrhage on foveal functional and morphologic prognoses, and to test the hypothesis against the effect of anti-VEGF agents on these prognoses, we studied 81 consecutive patients with or without subfoveal hemorrhage associated with acute BRVO.

## Methods

### Patients

The current study was approved by the Institutional Review Board (IRB) at Kyoto University Graduate School of Medicine, and adhered to the tenets of the Declaration of Helsinki. For this retrospective study, we reviewed the medical records of 81 consecutive patients (81 eyes) with acute BRVO, who were examined at the Department of Ophthalmology of Kyoto University Hospital between April 2009 and December 2014. According to our IRB guidelines, it was not mandatory to obtain informed consent from patients before retrospective reviewing of their medical records. Patient records were anonymized prior to analysis.

The inclusion criteria of this study were (1) symptomatic BRVO, in which retinal hemorrhage and retinal edema involved the macula, (2) foveal thickness of greater than 250 μm at initial visit as measured by OCT, (3) a duration of symptoms prior to the initial examination of less than 4 months, and (4) a minimum follow-up duration of 9 months, and a maximum follow-up duration of 20 months. The diagnosis of BRVO was based on fundus examinations and fluorescein angiography findings determined by four retina specialists (YM, AT, TM, SO). Eyes with central retinal vein occlusion (CRVO) or hemi-CRVO were not included in the current study. Eyes with co-existing ocular disease (*i*.*e*., AMD, retinitis pigmentosa, diabetic retinopathy, RMA, or senile cataract that resulted in poor image quality), and eyes that had any interventions for ME associated with BRVO before the study period were excluded.

### Examinations

At the initial examination, each patient underwent a comprehensive ophthalmologic examination which included measurement of best-corrected VA with a Landolt chart, and determination of intraocular pressure. Fundus biomicroscopy with a non-contact lens, 45° digital fundus photography (TRC-50LX; Topcon, Tokyo, Japan; 3216 × 2136 pixels), and OCT examination (Spectralis HRA+OCT, Heidelberg Engineering, Heidelberg, Germany) were performed after pupil dilation. To assess retinal perfusion status, each patient underwent fluorescein angiography with a confocal laser scanning system (HRA-2, Heidelberg Engineering, Heidelberg, Germany), or Optos 200Tx imaging system (Optos PLC, Dunfermline, United Kingdom). Eyes with BRVO were classified as ischemic when the area of nonperfusion was greater than 5 disc-diameters in size. The macular perfusion status was also classified as being complete or incomplete depending on the absence or presence of capillary dropout in the macular area.

Quantitative measurements and the other morphologic evaluations of the fovea were determined via the initial OCT images. Foveal retinal thickness was defined as the mean distance between the vitreoretinal interface and retinal pigment epithelium within a central subfield of the Early Treatment Diabetic Retinopathy Study (ETDRS) grid. Whether or not SRH was evident at the fovea via OCT images was determined. To assess the integrity of the foveal photoreceptor layer, we examined the conditions of the external limiting membrane (ELM) and ellipsoid lines within the central 2-mm area using vertical OCT sections centered on the fovea. The status of each was classified as being complete if the line could be completely detected, incomplete if the line was partially disrupted, or none if no line could be detected.

At each follow-up visit, each patient underwent a comprehensive ophthalmologic examination including measurement of best-corrected VA, color fundus photography, and OCT examination. We determined whether subfoveal hemorrhage was evident on the OCT sections obtained at each visit, and the duration of subfoveal hemorrhage. Using color fundus photographs obtained at the final visit, we performed a qualitative evaluation of the foveal appearance; specifically, whether it was intact or degenerative. Using the final vertical OCT sections centered on the fovea, we measured the foveal retinal thickness, and the defect length in the ELM or the ellipsoid line within the central 2-mm area. These OCT measurements were performed with the software built into the Spectralis HRA+OCT.

### Treatments

After August 2013, when ranibizumab was approved for BRVO-associated ME in Japan, 43 of the 81 eyes included in this study were treated with IVR [the IVR(+) group]. Each eye initially received three monthly IVR treatments, and was examined at our clinic every month. Additional injections were performed when ME and/or serous retinal detachment was evident at the fovea on OCT sections. The mean number of IVR injections was 4.5 ± 1.7. These eyes received no other treatment for BRVO, such as scatter laser photocoagulation, grid laser photocoagulation, steroid treatment, surgical intervention, or anti-VEGF agents other than ranibizumab.

Thirty-eight of the 81 eyes included in this study did not receive IVR [the IVR(-) group]. Sixteen eyes in the IVR(-) group underwent scatter laser photocoagulation for the nonperfusion areas outside the vascular arcades. None of these 38 eyes received treatments for ME such as laser photocoagulation within the vascular arcades, surgical intervention, injections of anti-VEGF agents, or steroid treatment.

### Statistical analysis

Statistical analysis was performed using PASW Statistics version 18.0 (SPSS, Chicago, IL). All values are presented as means ± standard deviation. For statistical analysis, VA measured with a Landolt chart was converted to a logarithm of the minimum angle of resolution (logMAR). Comparisons between the two groups were performed using the unpaired *t*-test, longitudinal changes within each group were analyzed using the paired *t*-test, differences in distributions were analyzed via chi-square tests, and bivariate relationships were analyzed via Pearson’s correlational coefficient. A stepwise forward multivariate linear regression analysis was performed to evaluate the contributions made by clinical factors to the foveal photoreceptor layers at final observation points. A *P* value of < 0.05 was considered statistically significant.

## Results

Table 1 shows initial measurements of all patients who were eligible for inclusion in this study. At the initial examinations, all eyes showed visual disturbance due to acute BRVO; mean VA was 0.42 ± 0.32, and mean foveal retinal thickness was 548.8 ± 165.5 μm. The macular perfusion status was judged as complete in 49 eyes, incomplete in 25 eyes, and undeterminable in 7 eyes because of blockage by a dense macular hemorrhage.

**Table 1:** Initial Clinical Characteristics of Eligible Patients with Acute Branch Retinal Vein Occlusion.

Number (persons/eyes)	81/81
Gender (men/women)	31/50
Age (years)	68.4 ± 11.4
Duration of visual disturbance from onset (months)	2.1 ± 1.4
Visual acuity (LogMAR)	0.42 ± 0.32
Foveal retinal thickness (μm)	548.8 ± 165.5
Subfoveal hemorrhage [eyes, (%)]	63 (77.8%)
ELM line at the fovea (complete/incomplete/none; eyes)	10/67/4
Ellipsoid line at the fovea (complete/incomplete/none; eyes)	10/66/5
Retinal perfusion status (perfused/nonperfused)	34/47
Macular perfusion status (eyes, complete/incomplete/undeterminable)	49/25/7

LogMAR = logarithm of the minimum angle of resolution; ELM = external limiting membrane.

### Morphologic manifestation of subfoveal hemorrhage associated with acute BRVO

**Fig 1:**
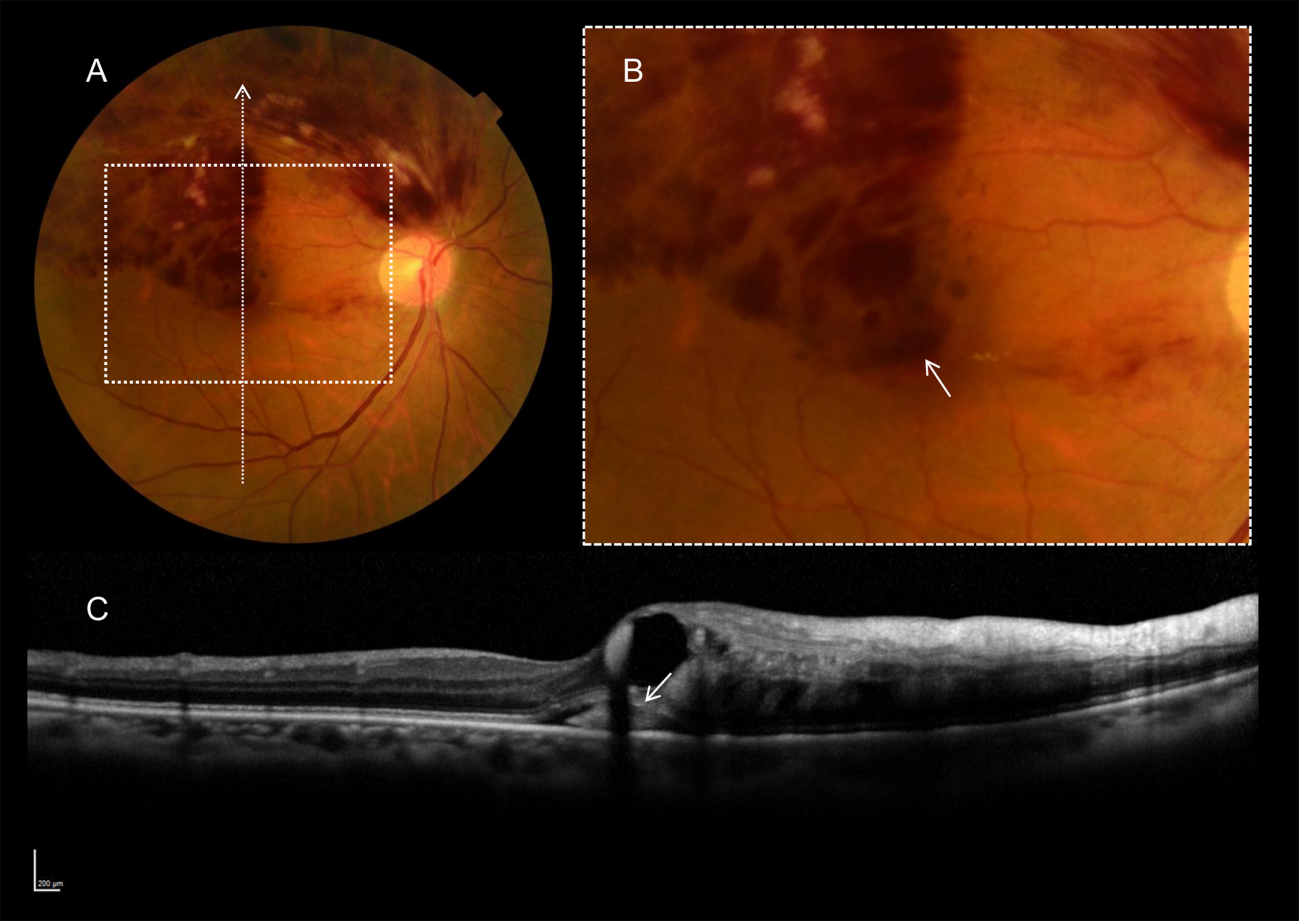
Subfoveal hemorrhage in eyes with BRVO. **Fundus photograph (A) and the magnified image (B) of an inset in (A), and the OCT image (C) corresponding to the arrow in (A).** On the color fundus photograph, subfoveal hemorrhage appears as a mat red lesion at the fovea (arrow in B). On the OCT section, subfoveal hemorrhage was detected as homogenous hyperreflective, or amorphous moderately hyperreflective material in the subretinal space (arrow in C).

In the current study, all 81 eyes with acute BRVO exhibited flame-shaped intraretinal hemorrhage in the affected retina, and ME involving the fovea (Fig 1). In addition, detailed observation revealed that 63 eyes (77. 8%) had subfoveal hemorrhage (Tables 1 and 2 and Fig 1). On color fundus photographs, most subfoveal hemorrhages appeared as a mat red lesion at the fovea. On OCT sections, subfoveal hemorrhages appeared as homogenous hyperreflective, or amorphous moderately hyperreflective material in the subretinal space (Fig 1).

**Table 2:** Initial and Final Findings in Eyes With or Without Subfoveal Hemorrhage Associated with Branch Retinal Vein Occlusion.

	Eyes without subfoveal hemorrhage 18 eyes	Eyes with subfoveal hemorrhage 63 eyes	*P*
Initial condition			
Visual acuity (LogMAR)	0.24 ± 0.30	0.47 ± 0.31	0.007
Foveal thickness (μm)	421.2 ± 153.3	586.4 ± 150.4	< 0.001
Duration of visual disturbance from onset (months)	2.0 ± 1.7	2.1 ± 1.4	0.622
Follow-up duration (months)	14.4 ± 3.5	13.4 ± 3.3	0.277
Final condition			
Visual acuity (LogMAR)	0.02 ± 0.17	0.21 ± 0.32	0.016
Foveal appearance (intact/degenerative; eyes)	20/0	25/38	< 0.001
Foveal thickness (μm)	286.1 ± 44.7	272.8 ± 53.5	0.338
Defect length in ELM line (μm)	0	489.4 ± 660.0	0.002
Defect length in ellipsoid line (μm)	0	594.6 ± 706.6	< 0.001

IVR = intravitreal injection of ranibizumab; LogMAR = logarithm of the minimum angle of resolution; ELM = external limiting membrane. *P* values are based on comparisons between the IVR(-) group and the IVR(+) group.

### Morphologic prognosis of eyes with subfoveal hemorrhage

Mean follow-up duration of our patients was 13.7 ± 3.4 months (S1 Table). At the final examinations, most eyes exhibited absorption of ME. Mean foveal retinal thickness was significantly reduced compared with the initial visit, to 275.7 ± 51.7 μm (*P* < 0.001), and there was also significant improvement in VA (*P* < 0.001).

**Fig 2:**
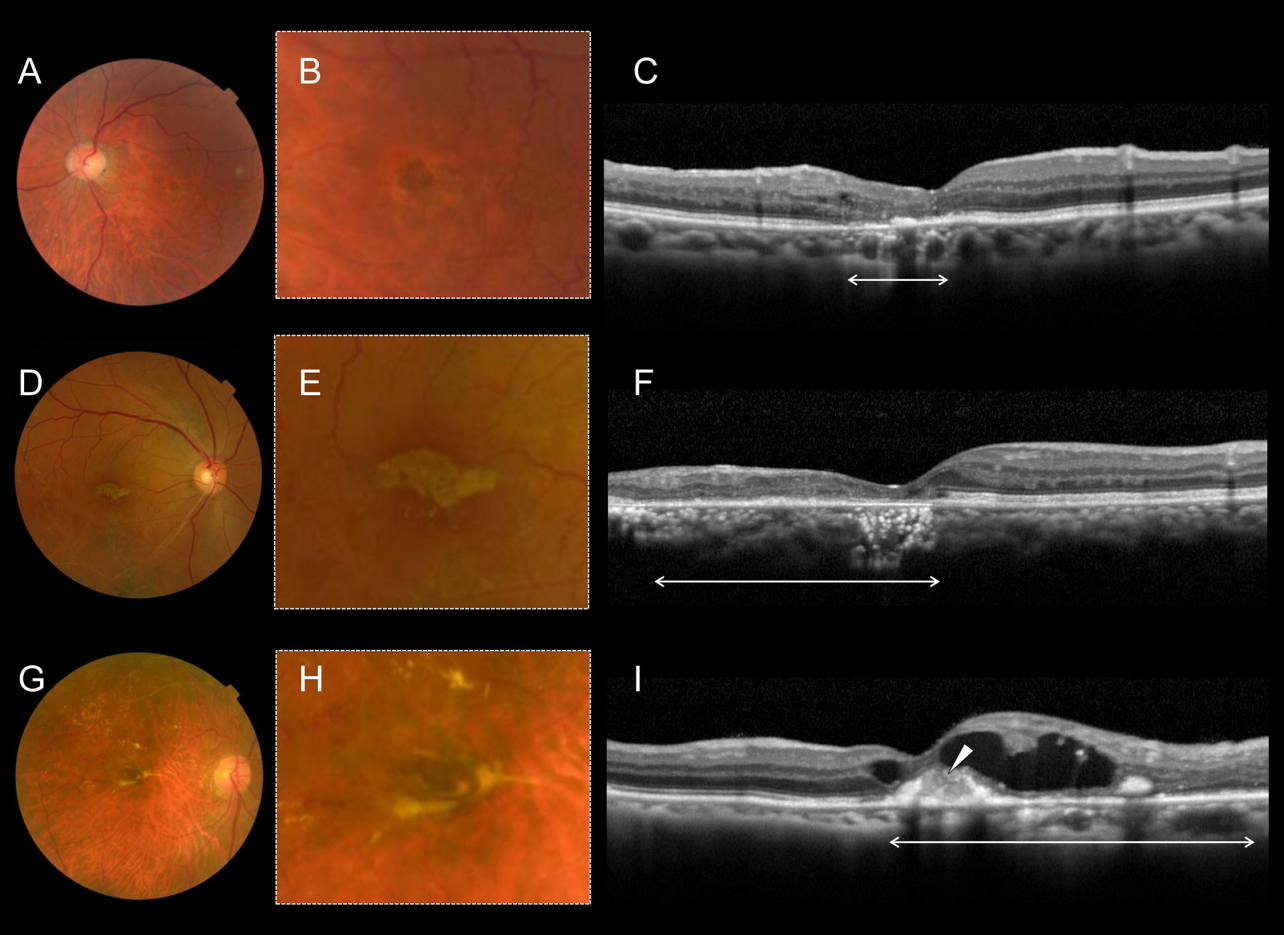
Foveal damage seen in three representative eyes with old BRVO. Color fundus photographs (A, D, G) and the corresponding magnified images (B, E, H) show gray-flat, or yellowish-fibrotic degeneration at the foveal areas of eyes with old BRVO. Consistent with the foveal degeneration seen in the fundus photographs, the vertical optical coherence tomography sections (C, F, I) of the foveal area show defects in the external limiting membrane and ellipsoid lines (arrows in C, F, I) or hyperreflective subretinal material (arrowhead in I).

**Fig 3:**
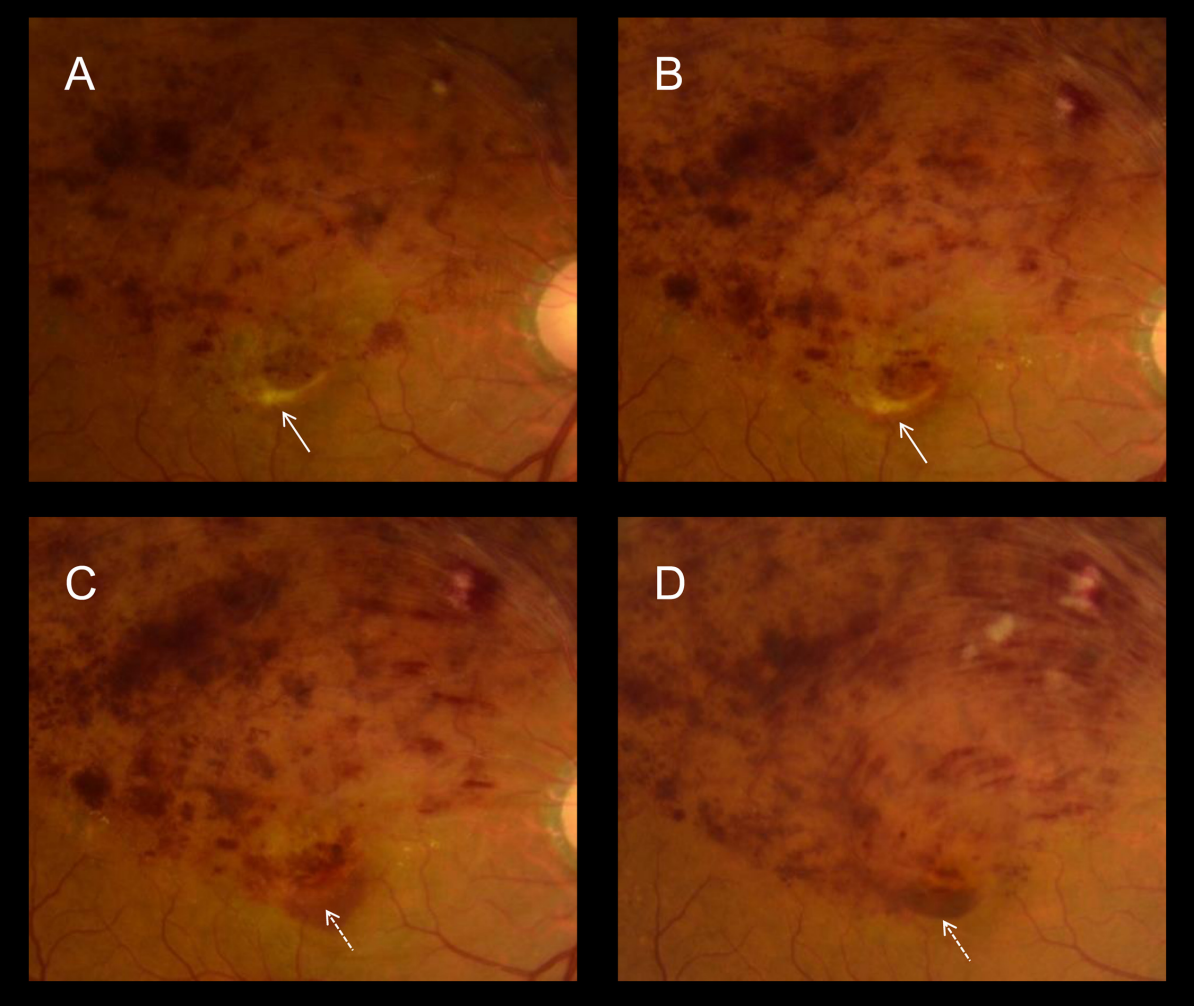
Formation process of foveal damage in an eye with BRVO accompanying subfoveal hemorrhage. Retrospective observation of color fundus photographs (A–D) demonstrates that the foveal areas of damage (solid arrows) are affected by the subfoveal hemorrhage seen in the acute phase of BRVO (dotted arrows).

**Fig 4:**
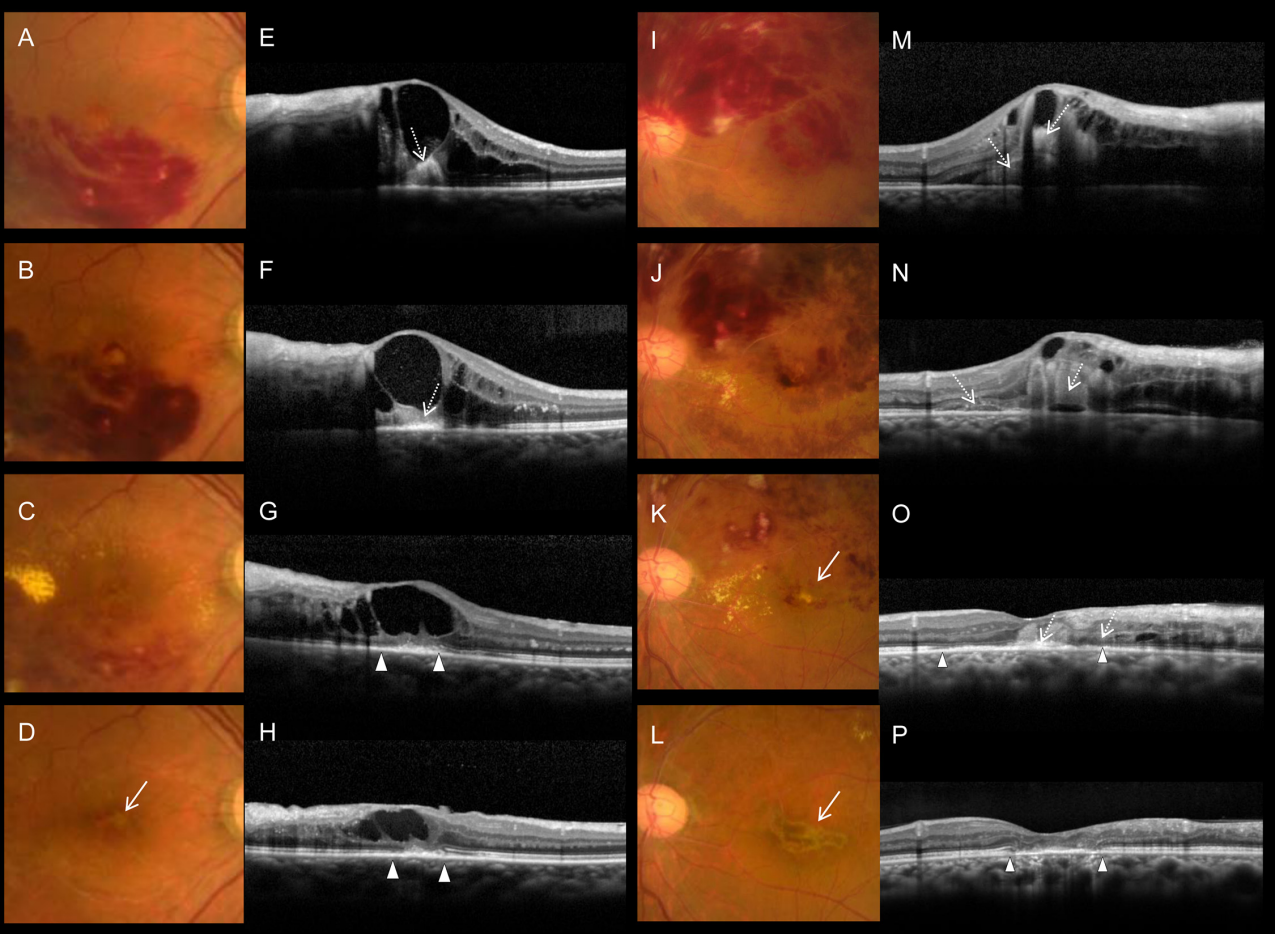
Foveal damage due to BRVO-associated subfoveal hemorrhage in two eyes not treated with ranibizumab. Fundus photographs (A–D) show BRVO in a 64-year-old female (follow-up period of 15 months). Retinal and subfoveal hemorrhages are completely absorbed (A–D), however, the final photograph (D) shows a mild degenerative change at the fovea (solid arrow in D). Vertical OCT sections of foveal areas (E–H) show that foveal and subfoveal hemorrhages are detected as homogenous hyperreflective, or amorphous moderately hyperreflective material in the outer retina and the subretinal space (dotted arrows in E, F), and the foveal external limiting membrane (ELM) and ellipsoid lines affected by the hemorrhage are lost (between the arrowheads in G, H). Initial and final visual acuity (Snellen equivalents) of the eye was 20/40, and 20/100, respectively. Fundus photographs (I–L) show BRVO in a 56-year-old male (follow-up period of 20 months). Color fundus photographs at chronic phase (K, L) show severe foveal damage has formed (solid arrow). Vertical OCT sections of the foveal areas (M–P) show that the foveal and subfoveal hemorrhages are detected in the outer retina and the subretinal space (dotted arrows in M, N), and the foveal ELM and ellipsoid lines, and consistent with that, the locations of the longstanding subfoveal hemorrhage are lost (between arrowheads in O, P). Initial and final visual acuity (Snellen equivalents) of the eye were 20/100 and 20/67, respectively.

In the final color fundus photographs, however, 38 (46.9%) eyes exhibited degeneration at the fovea (S1 Table); specifically, gray-flat, or yellowish-fibrotic degeneration was seen at the fovea (Fig 2). Consistent with the foveal degeneration evident in the fundus photographs, OCT sections revealed defective ELM and ellipsoid lines, or hyperreflective subretinal material (Fig 2). Retrospective evaluation of the foveal degeneration evident via color fundus photography revealed that the degenerative fovea of all such cases was afflicted with the subfoveal hemorrhage in the acute phase of BRVO (Fig 3). As shown in Fig 4 and S1 Fig, longitudinal OCT examinations revealed that damage to the foveal photoreceptor layers occurred in eyes with longstanding subfoveal hemorrhage, whereas persistent, or recurrent foveal edema did not always result in the damage of foveal photoreceptor layers (S2 Fig).

### Associations between functional prognosis and subfoveal hemorrhage

At the final examination, eyes without subfoveal hemorrhage exhibited better VA (0.02 ± 0.17), and none exhibited defective ELM or ellipsoid lines at the fovea. However, eyes with subfoveal hemorrhage often exhibited defective ELM and ellipsoid lines at the fovea at the final examination; the respective mean defect lengths were 489.4 ± 660.0 μm and 594.6 ± 706.6 μm, statistically significant increases in length for both parameters (*P* = 0.002 and *P* < 0.001, respectively). The final VA of eyes with subfoveal hemorrhage was significantly poorer (0.21 ± 0.32) than that of eyes without subfoveal hemorrhage (*P* = 0.016).

### Effect of IVR on visual prognosis

The mean age of the IVR(+) group was significantly greater than that of the IVR(-) group (*P* = 0.001, S2 Table), however, there were no significant differences in the other ocular parameters initially assessed, including VA, foveal retinal thickness, and duration of visual disturbance (S2 Table). At the final examination, while the difference in foveal retinal thickness was not statistically significant, final VA was significantly better in the IVR(+) group (*P* = 0.003, S2 Table). Additionally, foveal damage was significantly ameliorated in the IVR(+) group. With regard to the fundus photographs, foveal appearance was significantly better in the IVR(+) group (*P* = 0.006, S2 Table). Defects of the foveal ELM and ellipsoid lines apparent via OCT sections were significantly shorter in the IVR(+) group (*P* < 0.001 for both comparisons, S2 Table).

### Effects of IVR on subfoveal hemorrhage and other aspects of foveal pathomorphology

**Fig 5:**
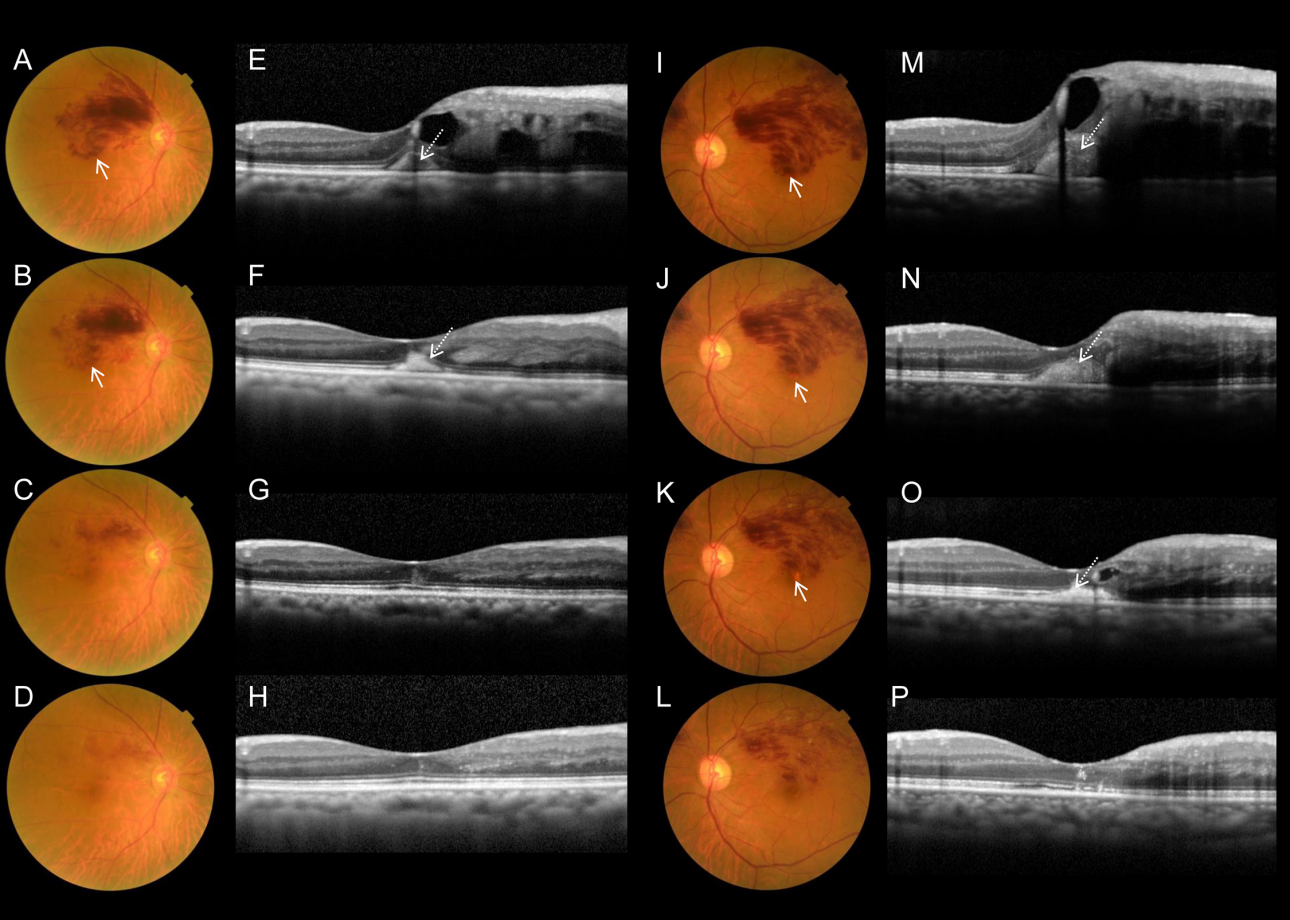
Minimal foveal damage in two eyes with BRVO-associated subfoveal hemorrhage that were treated with ranibizumab. (A–D, and I–L) Fundus photographs show BRVO with subfoveal hemorrhage (solid arrows) in an 81-year-old female, and a 65-year-old female respectively. (E–H, and M–P) OCT images of the foveal areas show the subfoveal hemorrhage is detected as homogenous hyperreflective material in the subretinal spaces (dotted arrows). (D, H and L, P) Each final photograph and OCT image is obtained just after the third IVR. IVR appears to accelerate the absorption of retinal hemorrhage, with rapid reduction of macular edema. Subfoveal hemorrhage is resolved quickly after the intervention. There are no foveal degenerative changes evident via fundus photography (D and L), and no defects or only minimal defects in the photoreceptor-layers are evident in the OCT sections (H and P). Initial visual acuity (Snellen equivalents) of the eyes were 20/66 and 20/40 respectively, and in both cases VA improved to 20/20 after IVR treatment.

Intraretinal hemorrhage and retinal edema associated with acute BRVO were absorbed gradually in eyes that did not undergo IVR treatment (Fig 4). However, IVR accelerated the absorption of intraretinal hemorrhage, in conjunction with a rapid reduction of ME (Fig 5). In addition, IVR seemed to accelerate the absorption of subretinal hemorrhage (Fig 5).

In eyes with no subfoveal hemorrhage, foveal morphology was well preserved with good visual prognosis in both the IVR(-) and IVR(+) groups (Table 3). In eyes with subfoveal hemorrhage, however, visual prognoses were more varied. To elucidate the effect of IVR on subfoveal hemorrhage and visual prognosis, we compared the final measurements in eyes with subfoveal hemorrhage in the IVR(-) and IVR(+) groups. There were no statistically significant differences in the initial ocular conditions tested, including VA, foveal retinal thickness, and duration of visual disturbance. With regard to the final examinations, while there was no significant difference in foveal retinal thickness, VA was significantly better in the IVR(+) group (*P* = 0.014). Foveal photoreceptor pathomorphology was highly preserved in the IVR(+) group; detection rates of the appearance of foveal degeneration were 75.0% in the IVR(-) group and 45.2% in the IVR(+) group (*P* = 0.016). In the IVR(+) group, defects in the foveal ELM line (236.8 ± 490.1 μm) and ellipsoid line (319.2 ± 541.6 μm) were significantly shorter than those of the IVR(-) group (734.1 ± 716.4 μm and 861.3 ± 751.9 μm, respectively; both *P* = 0.002). The duration of subfoveal hemorrhage was significantly shorter in the IVR(+) group (2.5 ± 4.2 months) than in the IVR(-) group (4.7 ± 3.2 months, *P* = 0.020). In some eyes that received IVR, subfoveal hemorrhage appeared to resolve quickly after the intervention despite any amount of the hemorrhage, and there were no foveal degenerative changes apparent via fundus photography, or defects in photoreceptor-layers evident in OCT sections (Fig 5).

**Table 3:** Initial and Final Findings in Eyes with Branch Retinal Vein Occlusion With or without Subfoveal Hemorrhage and IVR Therapy.

	Eyes without subfoveal hemorrhage			Eyes with subfoveal hemorrhage		
	IVR(-) group 6 eyes	IVR(+) group 12 eyes	*P*	IVR(-) group 32 eyes	IVR(+) group 31 eyes	*P*
Initial condition						
Visual acuity (LogMAR)	0.29 ± 0.45	0.22 ± 0.22	0.691	0.47 ± 0.30	0.48 ± 0.33	0.959
Foveal thickness (μm)	459.5 ± 136.0	402.1 ± 163.5	0.471	571.0 ± 135.7	603.3 ± 165.8	0.405
Duration of visual disturbance from onset (months)	2.6 ± 1.0	1.8 ± 1.9	0.376	2.3 ± 1.3	1.9 ± 1.4	0.216
Follow-up duration (months)	15.8 ± 4.2	13.8 ± 3.1	0.248	13.5 ± 3.7	13.3 ± 2.8	0.800
Final condition						
Visual acuity (LogMAR)	0.08 ± 0.21	-0.02 ± 0.14	0.288	0.31 ± 0.37	0.11 ± 0.24	0.014
Foveal appearance (intact/degenerative; eyes)	7/0	13/0	N.A	8/24	17/14	0.016
Foveal retinal thickness (μm)	309.8 ± 31.4	274.3 ± 46.7	0.114	269.3 ± 53.8	276.3 ± 53.8	0.609
Defect length in ELM line (μm)	0	0	N.A	734.1 ± 716.4	236.8 ± 490.1	0.002
Defect length in ellipsoid line (μm)	0	0	N.A	861.3 ± 751.9	319.2 ± 541.6	0.002
Duration of subfoveal hemorrhage (months)	0	0	N.A	4.7 ± 3.2	2.5 ± 4.2	0.020

IVR = intravitreal injection of ranibizumab; LogMAR = logarithm of the minimum angle of resolution; N.A = not applicable; ELM = external limiting membrane. *P* values are based on comparisons between the IVR(-) group and the IVR(+) group.

### Factors associated with visual dysfunction and foveal pathomorphology of eyes with old BRVO

**Fig 6:**
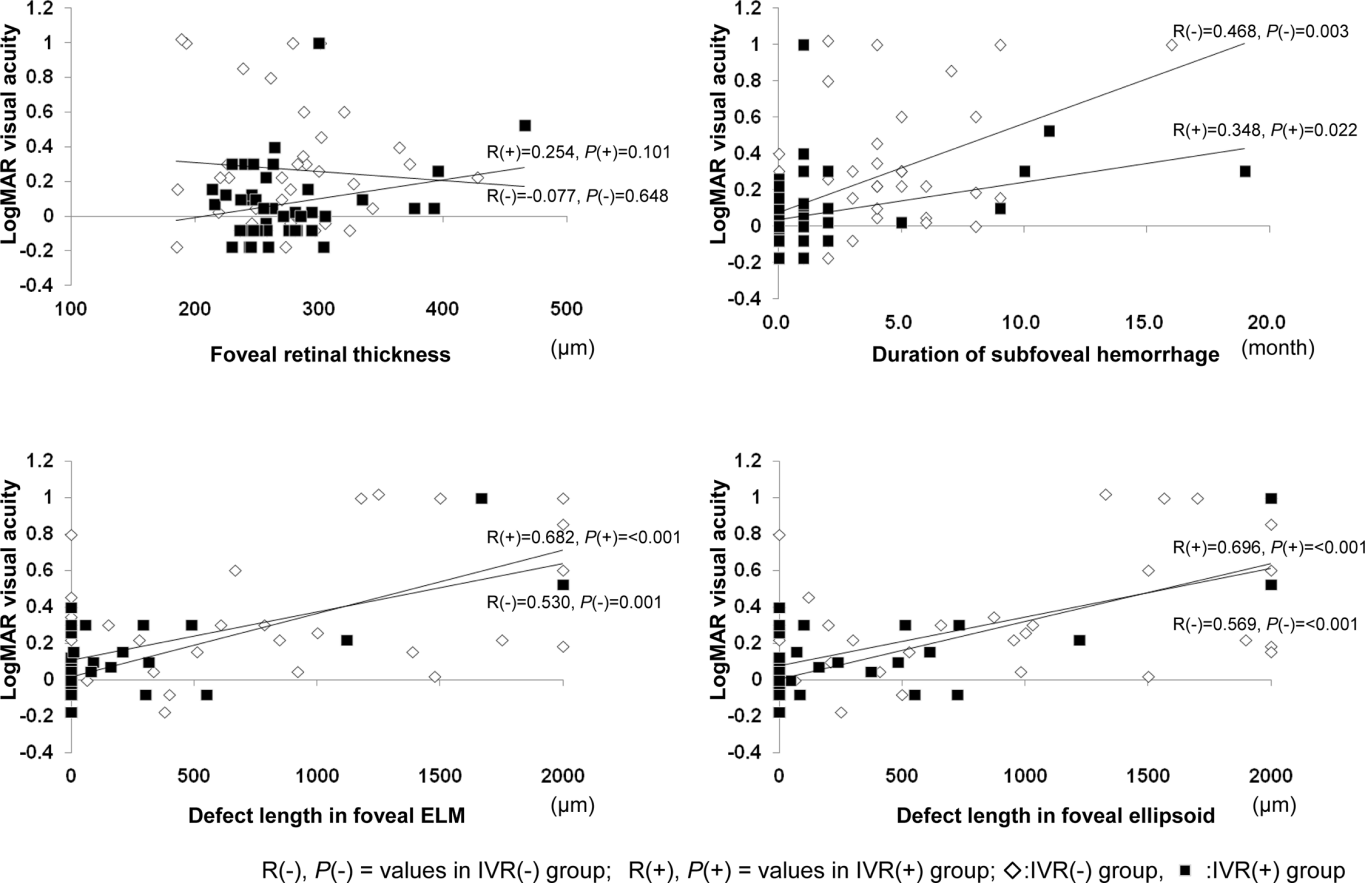
Associations between final visual acuity (VA) and other ocular parameters in the IVR(-) and IVR(+) groups. The scatter diagrams show the correlations between final VA foveal retinal thickness (A), duration of subfoveal hemorrhage (B), and defect lengths in foveal ELM (C), and ellipsoid lines (D) at the final visit. Final VA was not significantly correlated with final foveal retinal thickness in either group (A), however, it was significantly correlated with the length of the foveal defect in the ELM (C) and ellipsoid lines (D) in both groups (*P* < 0.001 in all cases). In addition, final VA was correlated with the duration of subfoveal hemorrhage (B) in the IVR(-) group (*r* = 0.468, *P* = 0.003) and in the IVR(+) group (*r* = 0.348, *P* = 0.022).

The associations between final VA and other ocular parameters in the IVR(-) and IVR(+) groups are shown below in Fig 6. Final VA was not significantly associated with final foveal retinal thickness in either group, but visual dysfunction was significantly associated with damage to the foveal photoreceptor layers in both groups. Final VA was correlated with the lengths of foveal defects of ELM and ellipsoid lines in both groups (*p* < 0.001 in both cases). In addition, final VA was significantly correlated with the duration of subfoveal hemorrhage in the IVR(-) group (*r* = 0.468, *P* = 0.003) and the IVR(+) group (*r* = 0.348, *P* = 0.022).

To comprehensively investigate the factors potentially associated with foveal degenerative changes, multivariate analysis was conducted in all subjects, in the IVR(-) group, and in the IVR(+) group, incorporating the following dependent variables: age, duration of visual disturbance from onset, follow-up duration, initial VA, initial foveal retinal thickness, and the duration of subfoveal hemorrhage of (Table 4). The analysis of the total subject population and the IVR(-) group showed that damage to the foveal photoreceptor layers was associated only with the duration of subfoveal hemorrhage. Of these independent variables; defect lengths in foveal ELM and ellipsoid lines were both closely correlated with the duration of subfoveal hemorrhage (total subjects: β = 0.568, *P* < 0.001 and β = 0.601, *P* < 0.001, respectively; IVR(-) group: β = 0.725, *P* < 0.001 and β = 0.770, *P* < 0.001, respectively). However, the association was not observed in the IVR(+) group; damage to the foveal photoreceptor layers was not associated with the duration of subfoveal hemorrhage (Table 4).

**Table 4:** Factors Associated with Foveal Photoreceptor Damage at the Final Visit in Eyes with Branch Retinal Vein Occlusion.

	Dependent values			
	Defect length in foveal ELM line		Defect length in foveal ellipsoid line	
	*β*	*P*	*β*	*P*
Total				
Age	-	-	-	-
Durations of visual disturbance from onset	-	-	-	-
Follow-up duration	-	-	-	-
Visual acuity at initial visit	-	-	-	-
Foveal retinal thickness at initial visit	-	-	-	-
Duration of subfoveal hemorrhage	0.568	< 0.001	0.601	< 0.001
IVR(-) group				
Age	-	-	-	-
Duration of visual disturbance from onset	-	-	-	-
Follow-up duration	-	-	-	-
Visual acuity at initial visit	-	-	-	-
Foveal retinal thickness at initial visit	-	-	-	-
Duration of subfoveal hemorrhage	0.725	< 0.001	0.770	< 0.001
IVR(+) group				
Age	0.121	0.453	0.071	0.680
Duration of visual disturbance from onset	-0.068	0.626	-0.065	0.664
Follow-up duration	-0.105	0.662	-0.013	0.960
Visual acuity at initial visit	0.227	0.175	0.325	0.077
Foveal retinal thickness at initial visit	0.704	< 0.001	0.625	< 0.001
Duration of subfoveal hemorrhage	0.271	0.280	0.194	0.467

The stepwise method was used in these multiple regression analyses. ELM = external limiting membrane, IVR = intravitreal injection of ranibizumab.

Finally, we investigated the effect of the initial macular perfusion status on the final VA. Table 5 shows the final measurement of the included eyes divided by the initial macular perfusion status. The final VA and foveal pathomorphology appeared to be better in the group with complete macular perfusion than in the group with incomplete macular perfusion, but the difference was not statistically significant.

**Table 5:** Final Ocular Measurements According to Initial Macular Perfusion Status of Eyes with Branch Retinal Vein Occlusion.

	Complete Macular perfusion at baseline (49 eyes)	Incomplete Macular perfusion at baseline (25 eyes)	P value
At final examination			
Visual acuity (LogMAR)	0.16 ± 0.32	0.22 ± 0.30	0.399
Foveal thickness (μm)	266.3 ± 44.8	285.0 ± 56.4	0.117
Defect length in ELM line (μm)	267.8 ± 502.2	475.5 ± 689.1	0.132
Defect length in ellipsoid line (μm)	332.8 ± 571.8	571.5 ± 731.2	0.111

## Discussion

Characteristic features often accompany acute BRVO, such as retinal hemorrhage, capillary nonperfusion, serous retinal detachment, or ME, some of which can cause impaired visual function.[1,2,4,20] The associations between macular ischemia and visual prognosis remain unclear. Finkelstein[2] reported that ischemic ME had a favorable visual prognosis in eyes with BRVO, but other investigators have reported poor visual function in eyes with the macular ischemia.[21] The association of serous retinal detachment with visual prognosis is also controversial although Ohashi et al. have reported that eyes with serous retinal detachment from BRVO had poor visual prognoses.[22] However, there is also no doubt that ME is significantly associated the visual impairment of eyes with BRVO, of which the introduction of anti-VEGF agents has improved the visual prognosis.[5,6,7,8]

In the present study, while the patients were in the acute phase, all affected eyes exhibited flame-shaped intraretinal hemorrhage. Such intraretinal hemorrhage seldom extends to the fovea because the fovea does not include the inner retina. However, eyes with acute BRVO frequently exhibit hemorrhage within cystoid spaces and/or subretinal spaces at the fovea. Muraoka et al. speculated that such hemorrhage was extravasated from the affected capillaries, and accumulated within the foveal cystoid spaces, then flowed into the subretinal space through the intraretinal break formed at the base of the cystoid spaces. In the current study, subfoveal hemorrhage was seen in 77.8% of cases. Detailed observation via OCT can facilitate the detection of the subfoveal hemorrhage associated with BRVO.

At the final examination of our patients, 46.9% exhibited degenerative fovea on color fundus photographs, and corresponding defects of the photoreceptor layers on OCT sections. Similar foveal degeneration has recently been reported in eyes with BRVO,[23] and CRVO.[24] In eyes with CRVO, the author speculated that they were associated with persistent ME and poor visual outcome.[24] In the current study however, we found no definite association between these foveal degenerations and the longstanding ME. Ota et al. reported that eyes with persistent ME after BRVO had good VA when the integrity of the outer aspect of the foveal photoreceptor layer was preserved.[9] In the current study, all such cases of foveal damage developed after longstanding subfoveal hemorrhage. Persistent subfoveal hemorrhage may cause overlying structural damage to the foveal photoreceptors, leading to a poor visual prognosis.

It is well recognized that submacular hemorrhage from neovascular AMD or RMA often causes severe visual impairment.[13,14,15,16,17,25] Previous experimental studies have suggested several mechanisms by which subretinal hemorrhage damages the overlying photoreceptors, such as clot retraction, iron toxicity (hemosiderosis), induction of fibrosis, and blockage of nutrient diffusion from the choroidal circulation.[18,19,26] In eyes with neovascular AMD accompanying subretinal hemorrhage, Notomi et al.recently reported an association between increased extracellular ATP and the neurodegenerative process.[27] In BRVO, some of these mechanisms may be involved in the formation of foveal damage, although the mechanisms involved are not completely understood.

In the natural course of acute BRVO, intraretinal hemorrhage and ME are absorbed gradually. However, IVR accelerates the speed of absorption of intraretinal hemorrhage, and is associated with a rapid reduction of ME. Pathologies of hemorrhagic lesions of the retina and brain indicate macrophages and microglia to phagocytose debris and red blood cells.[28,29] However, there has been no evidence that anti-VEGF agents modify these cellular functions. We can speculate that ranibizumab does not facilitate the absorption of intraretinal hemorrhage directly, rather, it has a suppressive effect on new bleeding seemingly accelerating absorption. In eyes with BRVO, ranibizumab undoubtedly improves visual function via the reduction of ME. Furthermore, ranibizumab may contribute to the improved visual prognosis, by indirectly accelerating the absorption of subfoveal hemorrhage, and subsequently mitigating damage to the overlying foveal photoreceptors.

In the current study, all eyes showed ME extending to the fovea, however, 22.2% did not exhibit accompanying subfoveal hemorrhage at the initial visit, and showed no defects of foveal ELM or ellipsoid lines at the final visit, and a mean final VA of 0.02 ± 0.17, with or without IVR treatment. This observation suggests that an absence of subfoveal hemorrhage is associated with a good visual prognosis. Recently, Zhao et al. reported the effect of intravitreal injections of bevacizumab for ME in 10 eyes with BRVO-associated subfoveal hemorrhage.[30] In their report, while bevacizumab did reduce macular thickness, this did not improve the visual outcome.[30] In the current study, even the eyes with subfoveal hemorrhage achieved VA improvement via IVR. The short follow-up period (6 months), small number of injections (a mean of 2.0), and low number of eligible eyes (*n* = 10) may explain the discrepancy in visual recovery.

One of the major limitations of the current study is its retrospective nature. The follow-up intervals and durations differed among the patients. Sixteen eyes in the IVR(-) group underwent scatter laser photocoagulation for the nonperfusion areas outside the vascular arcades, which may have resulted in a bias in the data. In addition, we could not evaluate changes in retinal nonperfusion areas. Its association with visual prognosis is therefore unclear.

However, despite these shortcomings, the assessment of 81 eyes with BRVO revealed that (1) subfoveal hemorrhage was not an uncommon feature, (2) visual and retinal morphologic prognoses of eyes without subfoveal hemorrhage were good, (3) the prognoses of those with subfoveal hemorrhage were poorer due to its association with damage to the overlying foveal photoreceptors, however (4) treatment with IVR could improve the visual and morphologic prognoses in such cases, by mitigating the disease process. Early treatment with IVR for BRVO-associated ME should be considered, to achieve better visual prognosis, especially in case with subfoveal hemorrhage.

## Supporting Information

S1 Fig Association between foveal damage and subfoveal hemorrhage, and the lack of association between the foveal damage and foveal detachment in an eye with BRVO. Longitudinal OCT reveals that damage to the foveal photoreceptor layers (between arrowheads in D) corresponds closely with the location of longstanding subfoveal hemorrhage (dotted arrows in A–C), whereas no damage is formed (solid arrow in D) at the locations of foveal detachment (arrowheads in B and C).
(TIF)

S2 Fig Weak association between persistent and recurrent macular edema (ME) and morphological changes in the foveal photoreceptor layers of an eye with BRVO. Longitudinal OCT with follow-up of 18 months (A–F) revealed that persistent and recurrent ME did not result in pathomorphological changes in foveal photoreceptor layers (F).
(TIF)

S1 Table Final Condition of Eligible Patients with Acute Branch Retinal Vein Occlusion (DOCX)

S2 Table Initial and Final Clinical Characteristics of Eligible Patients with Acute Branch Retinal Vein Occlusion in Groups Treated or Not Treated with Intravitreal Injections of Ranibizumab (DOCX)
