# Supplementary figures and images for: Foveal Damage Due to Subfoveal Hemorrhage Associated with Branch Retinal Vein Occlusion
Source: PLoS One. 2015 Dec 14;10(12):e0144894. doi: 10.1371/journal.pone.0144894 (PMC4677927; doi:10.1371/journal.pone.0144894)

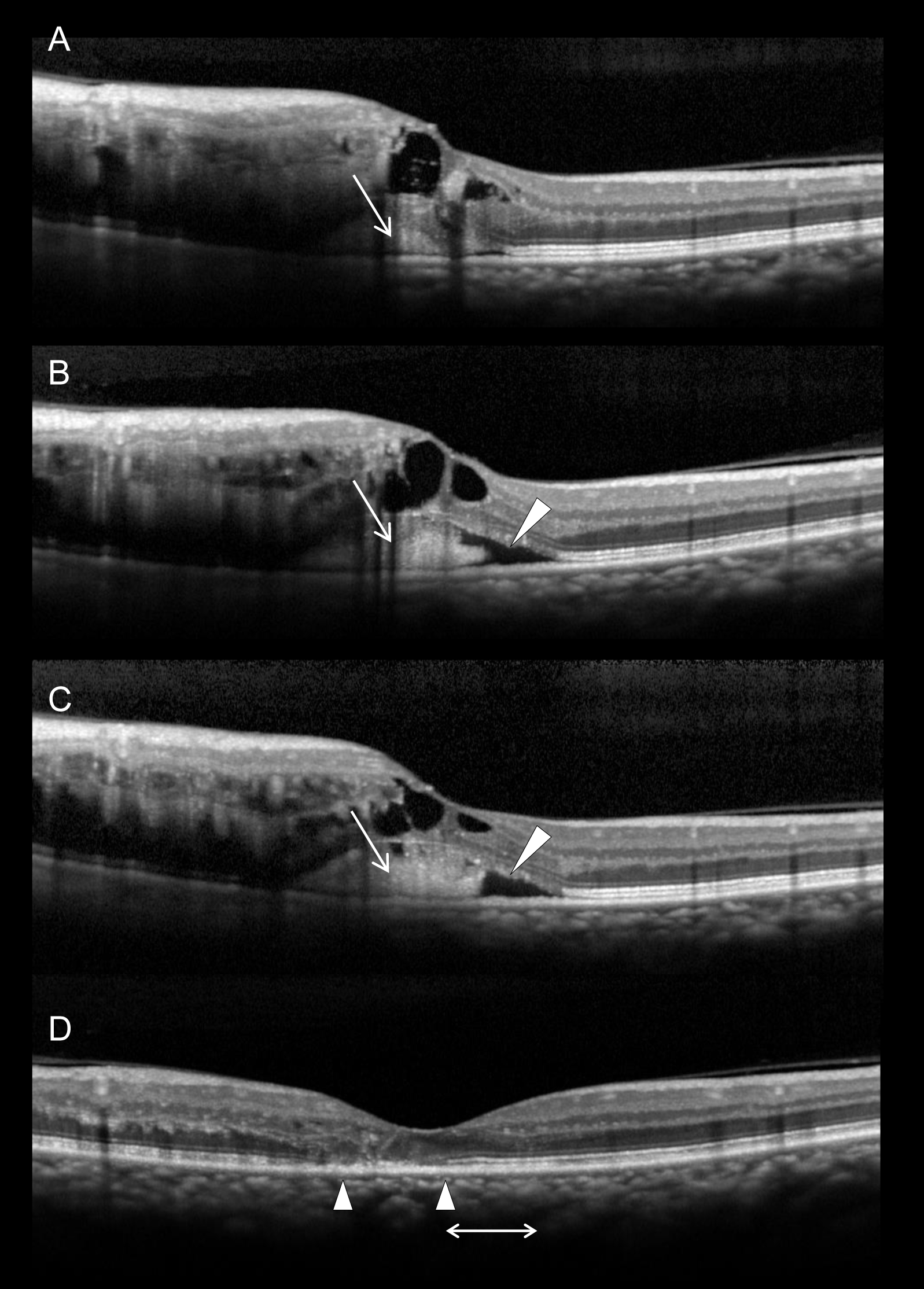

Supplement: S1 Fig — Longitudinal OCT reveals that damage to the foveal photoreceptor layers (between arrowheads in D) corresponds closely with the location of longstanding subfoveal hemorrhage (dotted arrows in A–C), whereas no damage is formed (solid arrow in D) at the locations of foveal detachment (arrowheads in B and C). (TIF) [file pone.0144894.s001.tif]

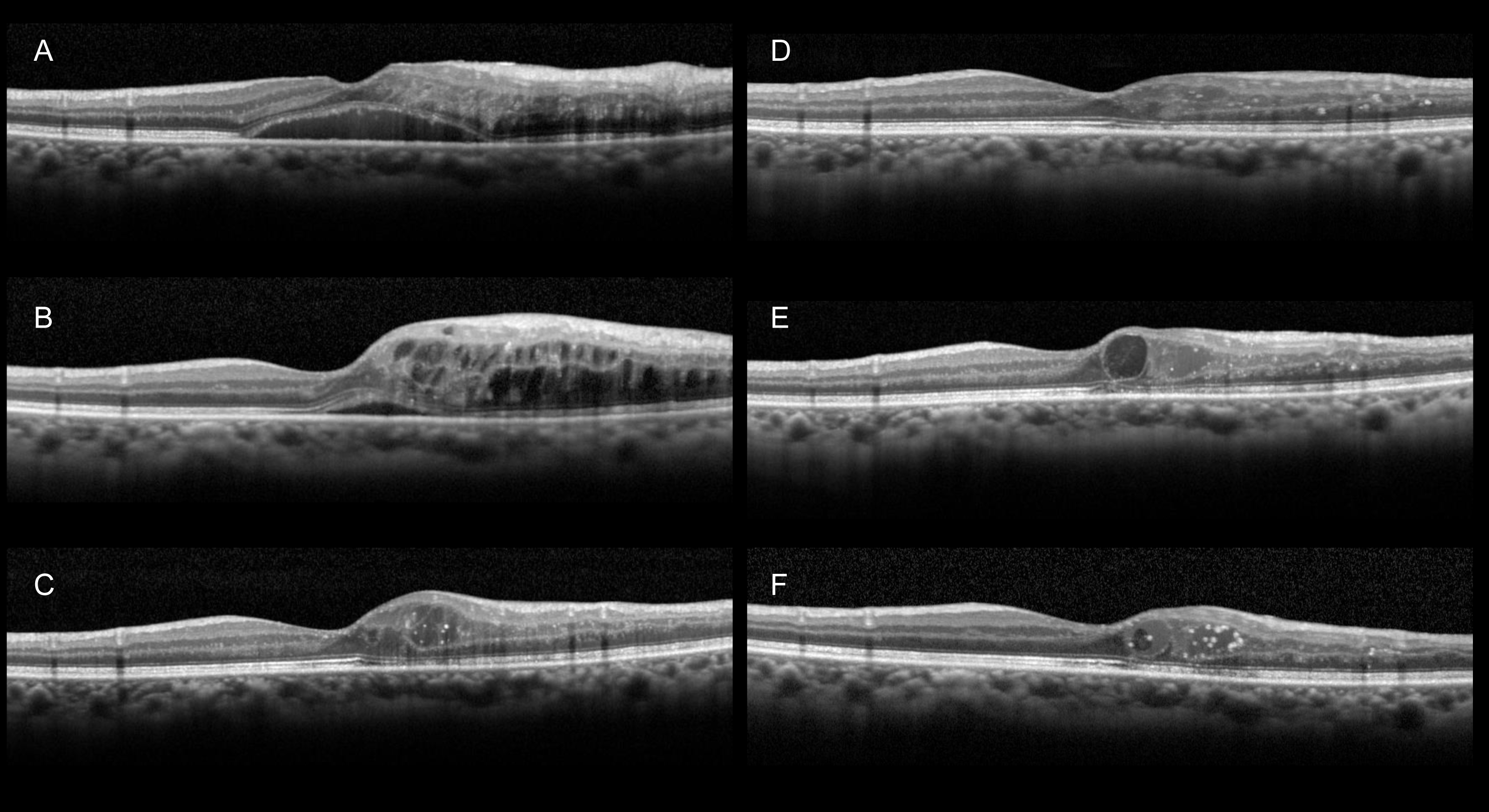

Supplement: S2 Fig — Longitudinal OCT with follow-up of 18 months (A–F) revealed that persistent and recurrent ME did not result in pathomorphological changes in foveal photoreceptor layers (F). (TIF) [file pone.0144894.s002.tif]
