# Supplementary material for: Foveal Damage Due to Subfoveal Hemorrhage Associated with Branch Retinal Vein Occlusion
Source: PLoS One. 2015 Dec 14;10(12):e0144894. doi: 10.1371/journal.pone.0144894 (PMC4677927; doi:10.1371/journal.pone.0144894)
Supplement: S1 Table — (DOCX) [file pone.0144894.s003.docx]

**Supplementary Table 1. Final Condition of Eligible Patients with Acute Branch Retinal Vein Occlusion**

| Follow-up duration (months) | 13.7 ± 3.4 |
| --- | --- |
| Visual acuity (LogMAR) | 0.17 ± 0.31 |
| Foveal appearance (intact/degenerative; eyes) | 43/38 |
| Foveal retinal thickness (µm) | 275.7 ± 51.7 |
| Defect length in foveal ELM line (µm) | 380.6 ± 616.0 |
| Defect length in foveal ellipsoid line (µm) | 462.4 ± 669.9 |

LogMAR = logarithm of the minimum angle of resolution; ELM = external limiting membrane.
