# Supplementary material for: Foveal Damage Due to Subfoveal Hemorrhage Associated with Branch Retinal Vein Occlusion
Source: PLoS One. 2015 Dec 14;10(12):e0144894. doi: 10.1371/journal.pone.0144894 (PMC4677927; doi:10.1371/journal.pone.0144894)
Supplement: S2 Table — (DOCX) [file pone.0144894.s004.docx]

| **Supplementary Table 2.** Initial and Final Clinical Characteristics of Eligible Patients with Acute Branch Retinal Vein Occlusion in Groups Treated or Not Treated with Intravitreal Injections of Ranibizumab | | | |
| --- | --- | --- | --- |
|  | IVR(-) group | IVR(+) group | *P* value |
| Number (persons/eyes) | 38/38 | 43/43 | N.A |
| Gender (men/women) | 18/20 | 13/30 | 0.113 |
| Initial condition |  |  |  |
| Age (years) | 64.3 ± 10.1 | 72.3 ± 11.3 | 0.001 |
| Duration of visual disturbance (months) | 2.4 ± 1.2 | 1.9 ± 1.6 | 0.121 |
| Visual acuity (LogMAR) | 0.44 ± 0.33 | 0.41 ± 0.32 | 0.609 |
| Foveal retinal thickness (μm) | 553.4 ± 140.1 | 544.5 ± 187.6 | 0.812 |
| Subfoveal hemorrhage [eyes, (%)] | 32 (84.2%) | 31 (72.0%) | 0.190 |
| ELM line at the fovea (complete/incomplete/none; eyes) | 2/34/2 | 8/33/2 | 0.190 |
| Ellipsoid line at the fovea (complete/incomplete/none; eyes) | 2/33/3 | 8/33/2 | 0.173 |
| Retinal perfusion status (nonischemic/ischemic) | 13/25 | 21/22 | 0.183 |
| Final condition |  |  |  |
| Follow-up duration (months) | 14.1 ± 3.8 | 13.5 ± 2.9 | 0.450 |
| Visual acuity (LogMAR) | 0.27 ± 0.36 | 0.11 ± 0.24 | 0.003 |
| Foveal appearance (intact/degenerative; eyes) | 14/24 | 29/14 | 0.006 |
| Foveal retinal thickness (µm) | 275.7 ± 52.8 | 275.7 ± 51.4 | 0.999 |
| Defect length in foveal ELM line (µm) | 618.2 ± 709.6 | 170.7 ± 427.9 | < 0.001 |
| Defect length in foveal ellipsoid line (µm) | 725.3 ± 758.3 | 230.1 ± 480.1 | < 0.001 |
| Abbreviations: IVR, intravitreal injection of ranibizumab; N.A, not applicable; LogMAR, logarithm of the minimum angle of　resolution; ELM, external limiting membrane. *p* values are based on comparisons between the IVR(-) group and the IVR(+) group. | | | |
